# Analysis of Processing Effects on Glucosinolate Profiles in Red Cabbage by LC-MS/MS in Multiple Reaction Monitoring Mode

**DOI:** 10.3390/molecules26175171

**Published:** 2021-08-26

**Authors:** Weicheng Wu, Jingqiu Chen, Dandan Yu, Shiguo Chen, Xingqian Ye, Zhiguo Zhang

**Affiliations:** 1 Food Science Institute, Zhejiang Academy of Agricultural Sciences, Hangzhou 311300, China; wuwc@zaas.ac.cn (W.W.); zhangzhg@zaas.ac.cn (Z.Z.); 2 College of Biosystems Engineering and Food Science, Zhejiang University, 866 Yuhangtang Road, Hangzhou 310027, China; 17816872926@163.com (J.C.); chenshiguo210@163.com (S.C.); psu@zju.edu.cn (X.Y.)

**Keywords:** glucosinolates, red cabbage, LC-MS/MS, MRM, cooking

## Abstract

Red cabbage (*Brassica oleracea* L. var. *capitata*) continues to receive increasing attention on its health-promoting properties because of its high glucosinolate content. Glucosinolates are an unstable active substance; however, there are few studies on their changes in different cooking processes. In this study, we investigated the effects of processing methods (boiling, steaming, microwave heating, frying, stir-frying) and boiling time on glucosinolates in red cabbage. Ten glucosinolates, including 4-methoxyglucobrassicin, neoglucobrassicin, glucoalyssin, glucobrassicin, glucoraphanin, glucoiberin, progoitrin, gluconapin and sinigrin, in red cabbage were detected. Decreases of 32.36%, 24.83%, 25.27%, 81.11% and 84.29% for total glucosinolates were observed after boiling, microwaving, steaming, frying and stir-frying. Indole glucosinolates were more efficiently lost compared to aliphatic glucosinolates after boiling, while microwaving, steaming, frying and stir-frying also resulted in a greater reduction in indole glucosinolates than aliphatic glucosinolates. Glucoalyssin, glucoerucin and sinigrin were more thermal sensitive than other glucosinolates. It was confirmed that microwaving and steaming retained higher levels of glucosinolates than other methods and may be better for cooking red cabbage.

## 1. Introduction

Red cabbage is popular worldwide and is beneficial due to its taste and intense red color, which increases the esthetic value of the food. It has received increasing attention because of its health-promoting properties, which are mostly ascribed to glucosinolates. Glucosinolates are a group of sulfur-containing secondary plant metabolites that are also known as β-D-thioglucoside-N-hydroxy sulfates, (Z)-(or cis)-N-hydroxyimino sulfate esters, or S-glucopyranosyl thiohydroximates. The side chain, R, is highly variable and, up to now, there has been more than 200 glucosinolates identified [1,2]. Red cabbage is mainly consumed by a variety of thermal cooking methods; these thermal treatments will further alter the glucosinolate content by mechanisms including enzyme breakdown by myrosinase upon cell lysis, thermal breakdown, and leaching into the heating medium [3,4,5]. Each cooking method involves specific conditions that lead to various degrees of impact on the glucosinolate content [6]. Both glucosinolates and their decomposition products exhibit important biological activities. In conventional cooking, such as steaming, heating begins at the surface of the food and heat is slowly transferred to the center by conduction. Conversely, in microwave cooking, microwaves permeate to the center of the food and the heat generated within the food is transferred toward the surface. In this respect, an equivalent rise in temperature occurs more quickly during microwave processing than steaming [7]. Hence, the effects of various cooking methods (i.e., boiling, steaming, microwaving, stir-frying or frying) and a broad range of boiling times on glucosinolate content in red cabbage were studied in this research.

Traditionally, gas chromatography (GC) was adopted for the identification and quantification of individual glucosinolates [8,9]. However, pre-column derivatization or conversion to volatile desulpho-glucosinolate derivatives has to be carried out, and some glucosinolates, particularly those containing indoles, are thermally unstable, making them unsuitable for GC [10]. Alternatively, high-performance liquid chromatography (HPLC) becomes the preferred analytical method. HPLC has the advantage of directly determining glucosinolates. In the application of identification and confirmation structures, it can be coupled to mass spectrometry (MS). MS has proved to be an invaluable tool in the identification and structural elucidation of glucosinolates. Hence, HPLC and MSn with electrospray ionization (HPLC-ESI/MSn) is widely used to identify the individual glucosinolates in plants [11,12]. However, most published methods for mass spectrometric analysis of glucosinolates required complex sample pretreatment [13,14]. Tian et al. developed a selective and sensitive quantitative method using liquid chromatography coupled to electrospray ionization tandem mass spectrometry with selected reaction monitoring detection, which can determine individual intact glucosinolates without conversion to desulfoglucosinolates [15]. SIM is selected ion monitoring, and it only selects the primary parent ion. MRM is multiple reaction monitoring, which selects the parent ion at first and then selects a daughter ion. Hence, the selectivity of MRM is higher than that of SIM [16].

In this study, we applied a rapid quantitative method for glucosinolate determination in red cabbage using high-performance liquid chromatography–electrospray ionization–tandem mass spectrometry (LC-ESI/MS/MS) with MRM, exhibiting superior sensitivity and selectivity. Ten individual glucosinolates in red cabbage were quantitatively determined. The method was first used to investigate the effects of processing methods and boiling time on glucosinolates in red cabbage and identified which glucosinolates were more stable during processing to determine the best cooking method.

## 2. Results and Discussion

### 2.1. Effect of Cooking Treatment on Proximate Composition in Red Cabbages

During cooking, various chemical and physical changes alter the proximate composition of red cabbage. Table 1 shows the proximate composition of the samples after being subjected to different cooking conditions. The results show that all cooking treatments increased crude fat but decreased crude protein and ash content. Boiling, microwaving and frying induced an increase in crude fiber, while the other two cooking methods, steaming and stir-frying, induced a decrease in crude fiber. The crude fat content of the stir-fried sample was much higher than those of raw or other cooked samples, mainly due to the absorption of fat by the red cabbage [17]. Murniece et al. obtained similar results to us, finding that the crude fiber content of deep-fat-fried samples was higher than in uncooked samples [18]. The reason suggested by Sun et al. (2014) was that frying caused structural damage to vegetable cells, leading to a significant loss in liposoluble constituents and a consequential increase in the fiber content [17]. The crude protein and ash content decreased to a greater extent after stir-frying (from 19.12 to 8.3 and from 7.68 ± 0.02 to 4.07 ± 7.53 g/100 g dry weight (DW)) than after applying the other cooking methods. Sun et al. (2014) found reductions in crude protein content after boiling, steaming, and microwaving, while frying increased crude fat content significantly, which was consistent with our results. The ash content decreased significantly after frying and stir-frying in our study. This result was in agreement with that reported by Gidamis et al. [19], who found a significant reduction in ash content as a result of cooking. The effects of boiling time on proximate composition in red cabbage were also determined (Table 2). When boiling time was increased from 0 to 30 min, a gradual decrease in ash content (from 7.68 ± 0.02 to 5.74 ± 9.14 g/100 g DW) and a gradual increase in crude fiber content (from 4.52 ± 2.46 to 13.28 ± 0.01 g/100 g DW) were observed. Although not appreciably changed, the crude fat content increased at first (6.77 ± 0.50 to 12.96 ± 0.16 g/100 g DW from boiling for 1 min), followed by a decreasing trend (12.96 ± 0.16 to 6.20 ± 0.25 g/100 g DW comparing boiling for 1 min to 30 min). In contrast, the crude protein content decreased at first, followed by an increasing trend and restoring to the original level after boiling for 20 min. Few data are available on the effect of boiling time on proximate composition, so the present study provides a reference for other researchers.

### 2.2. Identification of Individual Glucosinolate in Red Cabbage

**Figure 1:**
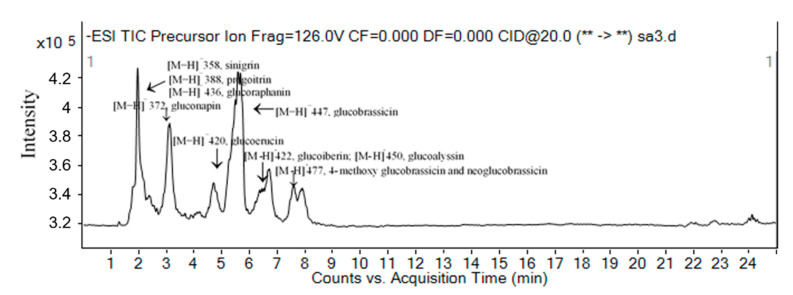
Total ion chromatogram (TIC): precursor ion scan of glucosinolates (molecular mass ions m/z [M−H]−) in red cabbage.

**Figure 2:**
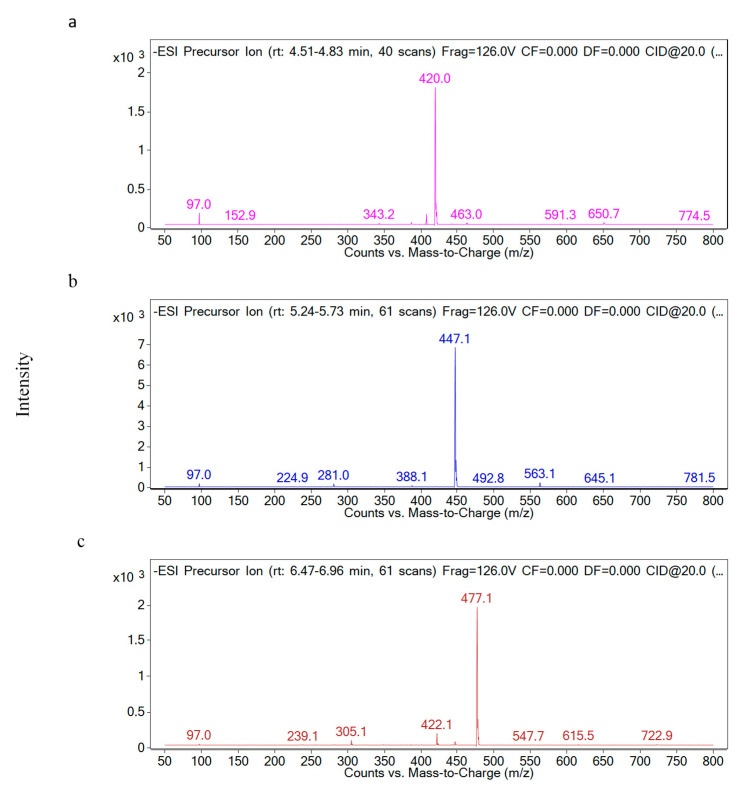
Molecular ion region of electrospray ionization mass spectra of: (**a**) glucoerucin [m/z 420, (M−H)−]; (**b**) glucobrassicin [m/z 447, (M−H)−]; (**c**) 4-methoxy glucobrassicin and neoglucobrassicin [m/z 477, (M−H)−].

The glucosinolate profile of red cabbage was analyzed by a total ion chromatogram (TIC) precursor ion scan (Figure 1). However, the peak signals for some glucosinolates (i.e., sinigrin, progoitrin and glucoraphanin) were overlapping in the TIC. All of the glucosinolates were identified by their [M−H]− ions and their molecular weight. The precursor ion scan revealed peaks at m/z 358, 372, 388, 420, 422, 436, 447, 450 and 477, representing sinigrin, gluconapin, progoitrin, glucoerucin, glucoiberin, glucoraphanin, glucobrassicin, glucoalyssin, 4-methoxy glucobrassicin and neoglucobrassicin, respectively, and Figure 2 shows data for typical glucosinolates, including glucoerucin, glucobrassicin, 4-methoxy glucobrassicin and neoglucobrassicin. This profile of the ten main glucosinolates found in red cabbage is in agreement with previous reports [3,20,21]. Additionally, 4-methoxy glucobrassicin and neoglucobrassicin are isomers that differ only in the position of a methoxy group. Hence, they were differentiated by reference to the reported elution sequence during reversed-phase HPLC [22]. Quantitative determination of glucosinolates in red cabbage can be achieved by a combination of adequate component separation and appropriate detection selectivity. The discriminating characteristic of LC-MS/MS using MRM detection is its superior sensitivity and selectivity. The negative ion MS/MS used in the present study showed that the MS/MS of the deprotonated glucosinolates parent ([M−H]−) generated a characteristic fragment with m/z 97 ([SO3H]−), facilitating MRM in the MS/MS studies. The MRM of the transition [M−H]− to m/z 97 was used to quantify each glucosinolates. Ten glucosinolates were determined simultaneously during the analysis, providing improved sensitivity and selectivity compared to selected ion monitoring techniques. Quantification was conducted according to the literature [15]. Molar absorption coefficients (λ = 235 nm) for sinigrin (εmol = 6780), glucoiberin (εmol = 6234), glucoerucin (εmol = 6531) and progoitrin (εmol = 4130 ± 135) were used to quantify. The linearity and precision of the method were evaluated within the dynamic range of interest. The UV absorbance of the glucosinolates was used to calculate their concentration [2].

### 2.3. Variation of Individual and Total Glucosinolate Content in Cooked Red Cabbage

Table 3 shows the individual and total glucosinolate content of red cabbage after different cooking treatments. The results reveal that after frying treatment, including frying and stir-frying, the total glucosinolate content decreased by 81.11% and 84.29%, respectively. Both microwaving and steaming produced smaller reductions in total glucosinolates compared to boiling (24.83%, 25.27% and 32.36%, respectively). Similar results were obtained by Verkerk et al. and Xu et al. [20,23]. As thermally unstable secondary plant metabolites, total glucosinolates levels were significantly reduced because of degradation into isothiocyanate, thiocyanate and nitrile at the high temperatures of frying and stir-frying. The aqueous solubility of glucosinolates is the reason that boiling caused more glucosinolate loss than steaming or microwaving. However, in the report by Verkerk et al. [20], the glucosinolate content was increased after microwave treatment. This may be due to differences in the red cabbage variety used, pretreatment and analysis methods. Therefore, systematic analysis of the effect of cooking methods on red cabbage is needed.

Glucosinolates are usually classified into aliphatic, aromatic and indole glucosinolates and only aliphatic and indole glucosinolates are present in red cabbage [1]. Aliphatic glucosinolates decompose into volatile isothiocyanates (responsible for pungent taste), whereas indole glucosinolates form indole compounds that are inhibitors of carcinogenesis [24].

#### 2.3.1. Effect of Cooking on Indole Glucosinolates in Red Cabbage

In red cabbage, glucobrassicin (7.1302 μmol/g DW) is the predominant glucosinolate, one of whose degradation products, 3-indolemethanol, can prevent mammary carcinogenesis [25]. Neoglucobrassicin and 4- methoxy glucobrassicin are also indole glucosinolates that are present in red cabbage. Frying caused the greatest reductions in glucobrassicin, neoglucobrassicin and 4- methoxy glucobrassicin (78.65%, 98.54% and 70.27%, respectively), followed by stir-frying (75.78%, 82.58% and 59.16%, respectively). Microwaving and seaming induced less reductions on glucobrassicin (19.63% and 13.39%, respectively) and neoglucobrassicin (6.33%, 17.65%, respectively) than boiling, whereas boiling for 3 min retained the highest 4- methoxy glucobrassicin content. On an overall level, red cabbage lost more indole glucosinolates than aliphatic glucosinolates after boiling for 3 min, showing the opposite effect to those of other cooking methods.

The breakdown products from indole glucosinolates have been identified as phytoalexins, which contribute to plant defense. In particular, 3-indolemethanol, because of its biological activity in the prevention of mammary carcinogenesis, is a potential chemo-preventive agent [26]. It is widely accepted that indole glucosinolates of Brassica vegetables are thermally less stable than aliphatic ones during heat treatment [1,27]. To retain the indole glucosinolate ingredient, the cooking method of microwaving is recommended for cooking red cabbage, followed by steaming.

#### 2.3.2. Effect of Cooking on Aliphatic Glucosinolates in Red Cabbage

Aliphatic glucosinolates exhibit a variety of biological activities in humans. There are four aliphatic glucosinolates that are found in relatively high levels in red cabbage, including glucoraphanin (1.3527 μmol/g), glucoerucin (1.3083 μmol/g), progoitrin (1.6057 μmol/g) and gluconapin (1.4480 μmol/g) (Table 3). Glucoraphanin is a precursor of the autism spectrum disorder treatment and anti-cancer compound, sulforaphane [28]. The anti-carcinogenic activity can be explained by the induction of Phase II detoxification enzymes or the inhibition of Phase I enzymes [29]. Glucoerucin is a precursor of 4-methylthiobutyl isothiocyanate, which is responsible for a radish-like flavor [30]. Good direct anti-oxidant activity of glucoerucin has been reported because of its ability to decompose hydroperoxides and hydrogen peroxide [31]. Goitrin, a degradation product of progoitrin, is anti-nutritional, toxic and goitrogenic [32]. However, as these goitrogenic compounds are all produced from chemically unstable isothiocyanates, the occurrence of these substances is dependent on enzymatic degradation, protein cofactors, and processing conditions. Further studies are needed in this respect. The anti-carcinogenic activity of progoitrin hydrolysis products has been reported by Cartea et al. [29]. Gluconapin is a precursor of 3-butenyl isothiocyanate, which is responsible for pungency and aroma [30]. However, knowledge on the effects of cooking on the thermal stability of aliphatic glucosinolates in red cabbage is scarce. It can be seen from Table 3 that frying and stir-frying resulted in excessive losses in glucoraphanin (82.06% and 83.94%, respectively), glucoerucin (92.48% and 98.26%, respectively), progoitrin (88.29% and 79.94%, respectively) and gluconapin (92.29% and 95.88%, respectively). Boiling, microwaving and steaming decreased glucoraphanin by 16.23%, 13.02% and 6.06%. Microwaved cabbage retained the highest gluconapin content, with only a 5.67% loss, followed by steaming (26.01%) and boiling (27.41%), while cabbage boiled for 3 mins retained the highest progoitrin content, with 3.81% loss, respectively. High thermolability of glucoerucin and progoitrin has been reported [7]. In the present study, boiling, microwaving and steaming caused less than a 27.41% reduction in glucoraphanin, progoitrin and gluconapin but more than 65.93% in glucoerucin reductions, proving glucoerucin to be more thermally sensitive than progoitrin.

Glucoalyssin, glucoiberin and sinigrin were present at low levels in red cabbage (0.8410, 0.6505 and 0.1358 μmol/g DW, respectively). Almost no research reported biological activity of glucoalyssin. Glucoiberin has been reported to be very thermolabile [33]. Hydrolysis products of glucoiberin have been reported to suppress carcinogenesis, protecting human and animal cells [29]. Sinigrin can produce volatile and bioactive 2-propenyl isothiocyanate, which is pungent, lachrymatory and has a bitter flavor, and 2-propenyl thiocyanate, which has horseradish and garlic-like properties [16,30,34]. Stir-frying and frying induced significant losses in glucoalyssin (93.37% and 92.24%, respectively), glucoiberin (95.86% and 92.36%, respectively) and sinigrin (96.61% and 96.39%, respectively), much like the effect on the glucosinolates mentioned earlier. Boiling retained the highest glucoiberin levels, with only a 5.38% decrease, followed by steaming and microwaving (19.51% and 25.92%, respectively). Boiling, steaming and microwaving induced significant losses in glucoalyssin (42.67%, 30.75% and 91.71%, respectively) and sinigrin content (35.27%, 91.75% and 93.89%, respectively). It can be seen that the effect of microwaving on the loss of glucoalyssin and sinigrin was obviously greater than the effect on glucoiberin.

#### 2.3.3. Effect of Boiling Time on Glucosinolates in Red Cabbage

As shown in Table 4, a gradual reduction in the total amount of three-indole glucosinolate content accounting for 55.84% of total glucosinolates was observed after boiling for 1 min (25.03%), 5 min (47.47%), 10 min (65.08%), 20 min (81.25%) and 30 min (83.81%), whereas aliphatic glucosinolate content decreased relatively slowly after boiling for 1 min (16.33%), 5 min (39.27%), 10 min (46.90%), 20 min (61.80%) and 30 min (69.24%,). This is somewhat in conjunction with Ciska and Kozlowska, who found that indole glucosinolates were more efficiently lost compared to aliphatic glucosinolates as a result of greater diffusion properties [33]. All detected glucosinolate content except for glucoraphanin, glucoiberin, progoitrin and gluconapin lost more than 50% after boiling for 10 min, and total glucosinolates decreased by 57.05%. Rosa and Heaney boiled shredded cabbage for 10 min (vegetable–water ratio 1:5), finding reductions in excess of 50%, which is similar to the findings in our study [35]. Glucoraphanin, glucoiberin and progoitrin showed less thermal sensitivity than other glucosinolates, whose content decreased slowly in the range from 1 min (6.79%, 4.66% and 0.32%, respectively) to 30 min (49.45%, 43.78% and 49.67%, respectively). Boiling time is important for glucobrassicin retention, and red cabbage boiled for less than 5 min kept nearly half of its glucobrassicins. It was likely that complete thermal inactivation had occurred in boiled red cabbage. The extent of hydrolysis of glucosinolates can be affected by myrosinase, which is partially or totally inactivated during heat treatment [21,36].

Thus, all cooking methods decreased the content of glucosinolates in red cabbage. To some extent, our results are in agreement with those found by Jon Volden et al. [21], who reported the effects of steaming and boiling on the glucosinolate content of red cabbage. Steaming resulted in high retention of glucosinolates, in line with that reported by Francisco et al. [37]. Glucosinolates are water-soluble compounds and are usually lost during conventional cooking because of leaching into surrounding water, enzyme breakdown, or thermal breakdown. Moreover, degradation events at a high temperature may also occur, leading to the formation of volatile compounds and other compounds [38]. Oerlemans et al. investigated the effect of thermal degradation in glucosinolates in myrosinase-deactivated red cabbage, finding that glucosinolates are relatively heat stable at temperatures <100 °C [3]. It is prudent to assume that a potential loss of glucosinolates is a function of leaching into the processing waters as well as the thermal cooking method [39]. Overall, we have provided comprehensive data on the effects of different cooking methods, providing a reference for other researchers.

## 3. Materials and Methods

### 3.1. Plant Materials

Red cabbages (*Brassica oleracea* L. var. *capitata*) were purchased from local agricultural markets (Hangzhou, China). Ten to fifteen cabbage heads were selected, removing the outer leaves and central core. The cabbage heads were chopped into 0.5 × 5–6 cm pieces. The chopped cabbages were randomly divided into four groups and were used as the raw material for the following cooking treatments. A 300 g sample was taken as a reference for fresh, unprocessed cabbage [40].

### 3.2. Cooking Treatments

The detailed cooking conditions are listed in Table 5. In the boiling process, 200 mL water was used with boiling times of 1, 3, 5, 10, 20 and 30 min. Microwave heating was conducted by placing cabbage leaves in a microwave oven at 1000 W for 3 min. Soybean oil (1 L) was heated to 190 °C in a fryer for frying cabbages. For stir-frying, soybean oil (5 mL) was used to cook cabbages in a wok. The cooking time was 3 min for all processes except frying, which was for 3 s. All samples were drained for 30 s, cooled quickly with liquid nitrogen and stored at −80 °C for further analysis. The excess oil after stir-frying and frying was removed with ether [23,41].

### 3.3. Determination of Proximate Composition

The proximate composition (crude fat, crude fiber, crude protein and ash) of the samples was determined according to the Association of Official Analytical Chemists (AOAC). Briefly, the crude fat content was determined by weighing red cabbage samples and extracting the crude fat with n-hexane using a Soxhlet apparatus (ISO 11085:2015). To determine the crude fiber content, a sample of red cabbage powder was boiled in 0.255 M sulfuric acid for 30 min, filtered, washed, boiled in 0.313 M sodium hydroxide, filtered, washed again, and dried at 130 ± 2 °C for 2 h (ISO 5498:1981). The crude protein content was determined by the Kjeldahl method using a nitrogen-to-protein conversion factor of 6.25 (ISO 20483:2013). The ash content (%) was determined by weighing red cabbage samples before and after heat treatment in a furnace (550 °C for 4 h) (ISO 6636-1:1986).

### 3.4. Determination of Glucosinolates

#### 3.4.1. Sample Extraction

Sample extraction was according to a previously reported procedure of extraction glucosinolates from freeze-dried vegetables with some modification [15]. In brief, each sample (40 mg) was extracted in 1.5 mL polypropylene-capped microcentrifuge tubes using 750 μL of 70% aqueous methanol, heated in a water bath for 10 min at 70 °C. After cooling in an ice bath, the extracts were centrifuged at 15,000× *g* for 15 min, and then the supernatants were removed using a syringe and filtered through a 0.22 μm nylon filter. The extraction procedure was repeated three times and the supernatants were combined. The supernatant was dried under nitrogen gas. The dried samples were reconstituted in 5 mL deionized water and filtered through 0.22 μm nylon filters for analysis.

#### 3.4.2. Analysis of Intact Glucosinolates

All mass spectra were obtained using an Agilent 6460 triple quadrupole ion-tunnel mass spectrometer coupled to a Waters 2696 HPLC system equipped with a 96-photodiode array detector. HPLC separation was performed on a 250 × 4.6 mm (5 μm) Luna C18 (2) reversed-phase column. A linear-gradient mobile phase that went from 100% A (water containing 0.5% trifluoroacetic acid) to 15% B (acetonitrile) in 10 min, to 40% B in 5 min, to 50% B in 5 min, and returned to 100% A in 5 min was used to elute the analytes at a flow rate of 1 mL/min. Approximately 100 μL/min of the HPLC eluate separated by a microsplitter was delivered to the Z-spray ESI source. Negative ion MS/MS was conducted to detect glucosinolates using MRM. The mass spectrometer was tuned by direct infusion of standard sinigrin, producing maximum abundant precursor ion m/z 358 ([M−H]−) and fragment ion m/z 97 ([SO_3_H]−) signals (approximately in equivalent abundance) during MS/MS. The mass spectrometric conditions were as follows: capillary, 2.35 kV; cone voltage, −35 V (RF-1, 50 V); desolvation gas temperature, 450 °C at a flow of 16.5 L/min; source temperature, 120 °C; collision energy, 18 eV. The following transitions were used to assay ten individual glucosinolates: glucoiberin (422 > 97), sinigrin (358 > 97), progoitrin (388 > 97), glucoerucin (420 > 97), glucoraphanin (436 > 97), gluconapin (372 > 97), glucoalyssin (450 > 97), glucobrassicin (447 > 97), neoglucobrassicin (477 > 97), and 4-methoxy glucobrassicin (477 > 97). A dwell time of 0.08 s was used for each transition. Standard calibration curves were established by triplicate analyses (seven concentration levels). For glucosinolates in red cabbage, quantification was according to Tian et al. [15].

## 4. Conclusions

Compared to previously reported methods such as HPLC, the LC-ESI/MS/MS analysis using MRM detection allows for the direct quantification of glucosinolates with improved sensitivity and selectivity. This method has been successfully applied to the detection of individual glucosinolates in Brassica vegetables in past repots. This is the first use of the LC-ESI/MS/MS method for the determination of glucosinolates in red cabbage, and ten individual glucosinolates have been detected.

This study found that red cabbage was a rich source of glucosinolates based on the structural diversity and total concentration of glucosinolates present. Glucobrassicin was the predominant glucosinolate found in red cabbage. The glucosinolate content was significantly influenced by cooking. We also confirmed that microwaving and steaming retained higher levels of glucosinolates than other methods and had less of an effect on proximate composition in red cabbages; thus, they may be better for cooking red cabbage. However, further research on degradation products, extract in cooking water/oil and degrading enzyme (myrosinase) in red cabbage during cooking is still needed.

## Figures and Tables

**Table 1:** Effects of different cooking methods on proximate composition of red cabbage.

Poximate Composition	Thermal Processing Methods					
	Raw	Boiled	Steamed	Microwaved	Fried	Stir-Fried
Crude fat	6.77 ± 0.50 ^a^	11.61 ± 0.16 ^c^	7.49 ± 0.82 ^a^	9.78 ± 0.19 ^b^	13.14 ± 0.32 ^d^	43.59 ± 0.25 ^e^
Crude fiber	4.52 ± 2.46 ^c^	6.17 ± 8.94 ^e^	4.18 ± 8.54 ^b^	5.01 ± 0.05 ^d^	12.67 ± 0.07 ^f^	3.28 ± 7.21 ^a^
Crude protein	19.12	18.31	18.56	18.7	12.58	8.3
Ash	7.68 ± 0.02 ^e^	7.30 ± 8.08 ^d^	7.36 ± 2.49 ^e^	6.86 ± 0.01 ^c^	5.01 ± 7.49 ^b^	4.07 ± 7.53 ^a^

^a–f^ Significantly different at *p* < 0.05.

**Table 2:** Effects of boiling time on proximate composition of red cabbage.

Poximate Composition	Boiling Time				
	1 min	5 min	10 min	20 min	30 min
Crude fat	12.96 ± 0.16 ^e^	9.56 ± 0.24 ^d^	8.08 ± 0.16 ^c^	7.02 ± 0.25 ^b^	6.20 ± 0.25 ^a^
Crude fiber	4.93 ± 5.17 ^b^	7.78 ± 8.12 ^c^	7.92 ± 2.49 ^e^	8.02 ± 3.39 ^d^	13.28 ± 0.01 ^f^
Crude protein	18.13	18.50	19.05	19.12	19.56
Ash	7.52 ± 8.10 ^f^	7.19 ± 0.01 ^d^	6.49 ± 9.48 ^c^	5.51 ± 0.03 ^a^	5.74 ± 9.14 ^b^

^a–f^ Significantly different at *p* < 0.05.

**Table 3:** Effects of different cooking methods on on glucosinolate (μmol/g DW) in red cabbage.

Glucosinolates	Thermal Processing Methods					
	Raw	Boiled	Steamed	Microwaved	Fried	Stir-Fried
4-Methoxyglucobrassicin	1.2117	0.9107	0.8489	0.8112	0.4948	0.3602
Neoglucobrassicin	0.9424	0.7627	0.8827	0.7761	0.1642	0.0138
Glucoalyssin	0.8410	0.4827	0.5824	0.0697	0.0594	0.0558
Glucobrassicin	7.1302	4.2111	5.7305	6.1755	1.7267	1.5221
Glucoraphanin	1.3527	1.1332	1.2707	1.1766	0.2427	0.2172
Glucoiberin	0.6505	0.6155	0.5236	0.4819	0.0497	0.0269
Glucoerucin	1.3083	0.4457	0.1852	0.2768	0.0984	0. 0228
Progoitrin	1.6057	1.5446	1.3904	1.2824	0.1880	0.3221
Gluconapin	1.4480	1.0511	1.0714	1.3659	0.1116	0.0597
Sinigrin	0.1358	0.0879	0.0112	0.0083	0.0049	0.0046
Indole glucosinolates	9.2843	5.8845	7.4621	7.7628	2.3857	1.8961
Aliphatic glucosinolates	7.3420	5.3607	5.0349	4.6616	0.7547	0.6863
Total	16.6263	11.2452	12.4978	12.4244	3.1404	2.6112

**Table 4:** Effects of boiling time on glucosinolate (μmol/g DW) in red cabbage.

Glucosinolates	Boiling Time				
	1 min	5 min	10 min	20 min	30 min
4-Methoxyglucobrassicin	1.0952	0.8098	0.6141	0.1830	0.1530
Neoglucobrassicin	0.8160	0.6798	0.3969	0.2424	0.1571
Glucoalyssin	0.4830	0.1410	0.0644	0.0636	0.0380
Glucobrassicin	5.0497	3.3872	2.2315	1.3152	1.1926
Glucoraphanin	1.2608	1.0481	0.9157	0.7667	0.6838
Glucoiberin	0.6202	0.6088	0.5259	0.4519	0.3657
Glucoerucin	0.8487	0.2978	0.1630	0.1420	0.0442
Progoitrin	1.6005	1.2534	1.1940	0.9083	0.8082
Gluconapin	1.2120	1.0296	0.9996	0.4541	0.3087
Sinigrin	0.1172	0.0800	0.0363	0.0182	0.0092
Indole glucosinolates	6.9609	4.8768	3.2425	1.7406	1.5027
Aliphatic glucosinolates	6.1424	4.4587	3.8989	2.8048	2.2578
Total	13.1032	9.3354	7.1414	4.5458	3.7604

**Table 5:** Cooking conditions of different cooking processes.

	Temperature (Power)	Time	Material/Water (Oil)	Tool
Boiling	100 °C	3 min	300 g/2 L	Supor saucepan
Steaming	100 °C	3 min	None	FOTILE SCD20-01 steamer
Microwave heating	1000 W	3 min	None	FOTILE W25800-01AG microwave
Frying	160 °C	3 min	300 g/500 mL	Royalstar YSF458 wok
Stir-frying	190 °C	3 s	300 g/2 L	VESTA EF-81 frying pan
